# Ulcers of the Tongue from Decayed Teeth

**Published:** 1849-07

**Authors:** 


Ulcers of the Tongue from Decayed Teeth.—Ulcers of the tongue are often dependent on derangement of the digestive organs. This barometer of the stomach, as it has been called, becomes affected with sores; but these are removed on the proper condition and tone of the digestive organs being restored. Ulcers take place from the constant irritation of the tongue, as in old persons whose teeth are in a state of decay. A man becomes tired of having the stumps taken out; he is averse to submit to the pain it occasions; perhaps one or two wrecks of teeth stand up very sharp, the tongue is constantly grating against them, and thus ulceration is established. If the cause be not removed, if the ulcers are allowed to continue for some months, malignant action may take place, and the tongue become indurated.—*Liston's Lectures—Lancet.*

